# Adenosine A_2A_ and A_3_ Receptors as Targets for the Treatment of Hypertensive-Diabetic Nephropathy

**DOI:** 10.3390/biomedicines8110529

**Published:** 2020-11-23

**Authors:** Daniela Patinha, Carla Abreu, Carla Carvalho, Olga Mariana Cunha, Mariana Mota, Joana Afonso, Teresa Sousa, António Albino-Teixeira, Carmen Diniz, Manuela Morato

**Affiliations:** 1 Department of Biomedicine—Unit of Pharmacology and Therapeutics, Faculty of Medicine, University of Porto, 4200-450 Porto, Portugal; dspatinha@gmail.com (D.P.); joanavafonso@gmail.com (J.A.); tsousa@med.up.pt (T.S.); albinote@med.up.pt (A.A.-T.); 2 The Institute of Biomedical and Clinical Science, Medical School, University of Exeter, EX4 4QJ Exeter, UK; 3 LAQV@REQUIMTE, Laboratory of Pharmacology, Department of Drug Sciences, Faculty of Pharmacy, University of Porto, 4050-313 Porto, Portugal; carla_abreu3@hotmail.com (C.A.); carlaluisalcarvalho@gmail.com (C.C.); olga.m.cunha@hotmail.com (O.M.C.); marianacmota@gmail.com (M.M.); mmorato@ff.up.pt (M.M.); 4 MedInUP—Center for Drug Discovery and Innovative Medicines, University of Porto, 4200-319 Porto, Portugal

**Keywords:** diabetes, hypertension, diabetic complications, diabetic nephropathy, adenosine receptors

## Abstract

Diabetic nephropathy (DN) and hypertension are prime causes for end-stage renal disease (ESRD) that often coexist in patients, but are seldom studied in combination. Kidney adenosine levels are markedly increased in diabetes, and the expression and function of renal adenosine receptors are altered in experimental diabetes. The aim of this work is to explore the impact of endogenous and exogenous adenosine on the expression/distribution profile of its receptors along the nephron of hypertensive rats with experimentally-induced diabetes. Using spontaneously hypertensive (SHR) rats rendered diabetic with streptozotocin (STZ), we show that treatment of SHR-STZ rats with an agonist of adenosine receptors increases A_2A_ immunoreactivity in superficial glomeruli (SG), proximal tubule (PCT), and distal tubule (DCT). Differently, treatment of SHR-STZ rats with a xanthinic antagonist of adenosine receptors decreases adenosine A_3_ immunoreactivity in SG, PCT, DCT, and collecting duct. There is no difference in the immunoreactivity against the adenosine A_1_ and A_2B_ receptors between the experimental groups. The agonist of adenosine receptors ameliorates renal fibrosis, probably via A_2A_ receptors, while the antagonist exacerbates it, most likely due to tonic activation of A_3_ receptors. The reduction in adenosine A_3_ immunoreactivity might be due to receptor downregulation in response to prolonged activation. Altogether, these results suggest an opposite regulation exerted by endogenous and exogenous adenosine upon the expression of its A_2A_ and A_3_ receptors along the nephron of hypertensive diabetic rats, which has a functional impact and should be taken into account when considering novel therapeutic targets for hypertensive-diabetic nephropathy.

## 1. Introduction

Adenosine activates four specific membrane receptor subtypes: A_1_, A_2A_, A_2B_, and A_3_, and is particularly crucial for kidney function [1]. The adenosine A_1_ receptor modulates glomerular filtration rate (GFR) through contraction of the afferent arteriole and glomerular mesangial cells [1,2], regulates the tubular transport of electrolytes, and negatively regulates renin secretion [1,3]. The adenosine A_2A_ receptor dilates the efferent arteriole [1], mediates anti-inflammatory and immunosuppressive effects [4,5], stimulates renin release [1], and preserves the structure and function of podocytes [6]. Adenosine A_2B_ receptors mediate immunomodulatory and anti-inflammatory actions that contribute to tissue repair [7], while restraining mesangial cell growth [8] and promoting renal fibrosis and glomerulosclerosis, through IL-6 formation and local release of vascular endothelial growth factor (VEGF) and transforming growth factor-beta 1 (TFG-β1) [9,10]. Adenosine A_3_ receptor activation induces mesangial cell apoptosis [11] and exerts pro-fibrotic effects [5].

Diabetic nephropathy (DN) is the primary cause of end-stage renal disease (ESRD) [12]. It courses with glomerular hyperfiltration and hypertrophy, basal membrane thickening, and mesangial matrix expansion, leading to glomerulosclerosis, persistent proteinuria, and decreased GFR [13,14]. Hypertension independently contributes to DN [12], coexists in most diabetic patients [15], as well as represents a major cause of ESRD [16]. However, studies on DN rarely consider the coexistence of hypertension and diabetes. Early hyperfiltration predicts the development of ESRD [17], but the mechanisms underlying DN are still not fully characterized, although adenosine has been occasionally implied [7,18,19,20,21,22,23,24]. In particular, adenosine receptors have been implicated in renal fibrosis [25]. Kidney adenosine levels are markedly increased in diabetes [18], and the expression [22,26,27,28] and function [20] of renal adenosine receptors are altered in experimental diabetes. We previously showed that the adenosine A_2A_ receptor is downregulated in experimental diabetes associated with spontaneous hypertension [29]. However, there is no information on how endogenous or exogenous levels of adenosine regulate the expression of adenosine receptors in this context and what would be the functional relevance of that regulation. We aim at studying the role ascribed to adenosine (endogenous/exogenous) on the expression/distribution profile of renal adenosine receptors in spontaneously hypertensive rats (SHR) rendered diabetic by streptozotocin (STZ). We observe that endogenous adenosine promotes the expression of adenosine A_3_ receptors, while exogenous adenosine increases the expression of the adenosine A_2A_ receptor subtype. This adenosine receptor expression pattern might contribute to the overall protective role of adenosine in hypertensive diabetic nephropathy.

## 2. Experimental Section

### 2.1. Drugs

The following chemicals were bought from Sigma Aldrich (Sintra, Portugal): 2-chloroadenosine (CADO), 3,3-diaminobenzidine tetrahydrochloride (DAB), 1,3-dipropyl-8-sulfophenylxanthine (DPSPX), pentobarbital sodium salt, streptozotocin (STZ), and Triton X-100.

We used primary antibodies from Santa Cruz (Santa Cruz Biotechnology, Dallas, Texas, USA), namely rabbit polyclonal anti-A_1_, anti-A_2A_, anti-A_2B_, and anti-A_3_. Moreover, we worked with the rabbit biotinylated secondary antibody and the avidin-biotin complex reagents (ABC) from the Vectastain Elite ABC universal kit (Vector Laboratories, Burlingame, CA, USA). All reagents were of analytical grade.

Renal sections were stained with the Masson trichrome Goldner staining kit (Bio-Optica Milano Spa, Milano, Italy); urinary isoprostanes were quantified using an ELISA kit (Oxford Bio-medical Research Inc., Oxford, MI, USA), and H_2_O_2_ was measured using the Amplex Red Hydrogen Peroxide Assay kit (Molecular Probes, Eugene, OR, USA).

### 2.2. Animals and Treatments

Male spontaneously hypertensive rats (SHR; 12 weeks; Charles River, Barcelona, Spain) were used. Animals had free access to water and food and were housed under controlled conditions of temperature (22 °C), humidity (60%), and light-dark cycle (12 h/12 h). All experiments were performed in accordance with the European Union guidelines for the protection of animals used for scientific purposes (Directive 2010/63/EU). The protocols were in accordance with the ARRIVE (Animal Research: Reporting of In Vivo Experiments) guidelines for reporting experiments [30] and were approved by the Committee on the Ethics of Animal Experiments of the Faculty of Pharmacy of the University of Porto (Permit Number 13/11/2013).

On Day 0, diabetes was induced by an intraperitoneal injection of STZ (65 mg/kg) in all animals. After 48 h, blood glucose concentration was determined using an autoanalyzer (Abbott Diabetes Care Ltd., Santa Clara, CA, USA), and animals with a blood glucose concentration above 300 mg/dL were considered diabetic. On Day 14, animals were randomly assigned to three groups (*n* = 4 rats in each group). Animals from two groups were anesthetized with pentobarbital sodium (50 mg/kg; i.p.), and osmotic minipumps (Alzet model 2ML1; Alza, Palo Alto, CA, USA) were intraperitoneally implanted to allow continuous (10 mL/h) infusion of CADO (5 mg/kg/d; SHR-STZ+CADO group) or DPSPX (90 µg/kg/h; SHR-STZ+DPSPX group) during 7 days. CADO is a non-selective agonist of adenosine receptors [31] and DPSPX is a non-selective antagonist of adenosine receptors [32]. The animals allocated to the third diabetic group underwent a sham operation procedure and were taken as the control group of the study (SHR-STZ group). On Day 21, twenty-four hour urine samples were collected, and all animals were anesthetized as previously described. Blood samples were collected under anesthesia from the left ventricle to heparinized or EDTA ice-cold tubes. After centrifugation of blood samples (3000 rpm, 15 min, 4 °C), plasma was collected. All samples were stored at −80 °C until assayed. Simultaneously, the left kidney was excised and processed for histological analysis and immunohistochemistry. With this, the death of the anesthetized animals was ensured by exsanguination.

### 2.3. Metabolic Parameters, Renal Function, and Blood Pressure

Glucose and creatinine concentrations were determined in urine and plasma samples by a glucose oxidase method and the colorimetric Jaffé method, respectively. Total urinary protein concentration was determined using pyrogallol red. Na^+^ concentration was measured using ion-selective electrodes. These assays were performed using a Cobas Mira Plus analyzer (ABX Diagnostics, Geneva, Switzerland). Because high glucose concentrations interfere with the creatinine Jaffé method, creatinine concentration values were corrected using the method previously reported by Moreira-Rodrigues and co-workers [33]. The glomerular filtration rate (GFR) was calculated using the formula GFR = [Ucreatinine] × V/[Pcreatinine], where [Ucreatinine] and [Pcreatinine] denote the corrected creatinine concentration in the urine and plasma samples, respectively, and V denotes the urine flow rate (mL/min). Fractional Na^+^ excretion (FE_Na_) was calculated using the formula [UNa] × [Pcreatinine]/[PNa] × [Ucreatinine], where [PNa] and [UNa] represent plasma and urinary Na^+^ concentrations. On Day 18, animals where anaesthetized as described above, and an intra-arterial polyethylene catheter (PE10 connected to PE50; Bilaney, Dusseldorf, Germany) was placed on the aorta via the left femoral artery, passed subcutaneously using a trocar, and externalized at the back of the neck, where it was secured to the skin of the animal [20]. Catheters were daily flushed with saline heparinized solution (0.1%) to prevent clotting. At the end of the study, the catheter was connected to a pressure transducer (B. Braun, Bethlehem, PA, USA) coupled with a polygraph (Letica Polygraph 6006; Letica, Barcelona, Spain) to measure intra-arterial systolic blood pressure (SBP) in conscious, unrestrained animals.

### 2.4. Immunohistochemistry

The kidneys were fixed in 4% formaldehyde overnight, dehydrated in a graded series of ethanol followed by benzoyl, and embedded in paraffin. Sequential 4 μm thick renal sections were obtained from each animal and mounted onto poly-L-lysine slides. Experiments were carried out in five batches using five levels, corresponding to 300 kidney sections for the SHR-STZ, SHR-STZ + CADO, and SHR-STZ + DPSPX groups.

Immunohistochemistry was performed as previously described [29,34,35] with some modifications. Briefly, sections were dewaxed with toluene and rehydrated in a graded series of ethanol at room temperature (RT). Endogenous peroxidase was blocked using 3% H_2_O_2_ solution, and non-specific protein binding was blocked with 2% albumin in phosphate-buffered saline (PBS (g/L): 8 g NaCl; 0.201 g KCl; 0.191 g KH_2_PO_4_; 0.765 g Na_2_HPO_4_.2H_2_O). The following primary antibodies (Santa Cruz Biotechnology) were used: rabbit polyclonal anti-A_1_ (1:50 dilution), anti-A_2A_ (1:250 dilution), anti-A_2B_ (1:50 dilution), and anti-A_3_ (1:250 dilution), diluted in PBT (0.3% Triton X-100 in PBS). The specificity of the primary antibodies used in this study was previously tested by immunoprecipitation of the protein or knockdown using siRNA [36,37,38,39] and by pre-adsorbing individual primary antibody with a tenfold excess of its respective blocking peptides in SHR kidney samples [35].

Incubation with individual adenosine receptor primary antibodies was done overnight, at 4 °C, in a humidified chamber. Sections were then washed in PBT and incubated with a biotinylated anti-rabbit secondary antibody (1:125 dilution in PBT) for 1 h, at RT. Sections were then rinsed in PBT and incubated with avidin-biotin complex reagent (ABC) for 1h, at RT. After washing with PBS, sections were incubated with 3,3-diaminobenzidine tetrahydrochloride (DAB) activated with H_2_O_2_, which was used as a chromophore. Tissue sections were then washed with distilled water, dehydrated in a graded series of ethanol and xylol, and mounted with the Eukitt^®^ mounting medium.

**Figure 1:**
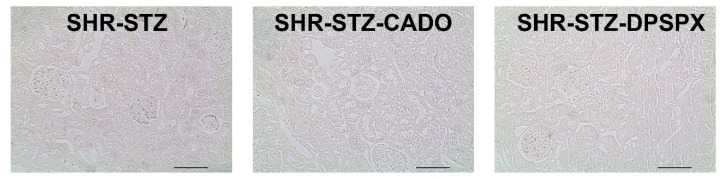
Secondary antibody controls for immunohistochemistry experiments: Representative photomicrographs of kidney transversal sections from spontaneously hypertensive rats rendered diabetic with streptozotocin with no further pharmacological treatment (SHR-STZ) and continuously infused with 2-chloroadenosine (CADO) (non-selective agonist of adenosine receptors) or 1,3-dipropyl-8-sulfophenylxanthine (DPSPX) (non-selective antagonist of adenosine receptors), showing the absence of staining both in glomeruli and in tubular structures. Negative controls were incubated in parallel using 10% normal horse serum or blocking solution instead of the primary antibody in order to determine the background level due to nonspecific secondary antibody binding. Note the clean background obtained in all sections from the three groups of animals in the study. Bars: 50 µm.

Negative control samples for non-specific binding of secondary antibody of kidney sections from SHR-STZ, SHR-STZ-CADO, and SHR-STZ-DPSPX were processed in parallel with the test sections using 10% serum (Vectastain Elite ABC kit; Vector Laboratories, Burlingame, CA, USA) instead of the primary antibody (Figure 1).

### 2.5. Imaging

Digital images from each immunostained section were acquired using a Digital Color Camera (Leica DFC295, Leica Mycrosystems, Carnaxide, Portugal) mounted on a Nikon Eclipse E400 microscope (Nikon Corporation, Tokyo, Japan) using LAS software (Leica Application Suite V3.5.0, Carnaxide, Portugal). All sections (including negative control sections) were analyzed under the same light conditions of brightfield optics, as well as camera exposure times. Acquired images (24 bit, 8 bit/color) from tissue sections had a resolution of 3072 × 2304 pixels, corresponding to 655 × 491 µm on the original histological section (1 pixel = 0.21 µm) with the calibration micrometer slide being adjusted to convert pixels into micrometers.

### 2.6. Histomorphometry

Digital images (RGB) from DAB-immunostained sections were assessed by quantitative histomorphometry using the PAQI software (CEMUP, Porto, Portugal), as previously described [35,40,41]. Briefly, color images from DAB-immunostained sections (immunostained with anti-A_1_, anti-A_2A_, anti-A_2B_, or anti-A_3_ antibodies) were converted into their blue component, and the following renal structures were isolated: superficial (SG) and deep (DG) glomeruli, proximal (PCT), distal (DCT), and collecting (CT) tubules, and loop of Henle (LH). Image analysis was used to set thresholds for automated DAB-staining segmentation. To determine differences between stained and non-stained tissue, control samples were imaged with the same microscope illumination and camera operating conditions, and the average of stained level was determined. This average value (171 for a maximum of 255) was used for threshold segmentation of the stained areas of each kidney structure. The level of immunohistochemical staining was obtained by quantifying the fraction of tissue stained with DAB (stained fractional area) using digital images of DAB-labeled immunostain from kidney sections. Histomorphometric analysis was previously described as a valid methodology and can be as effective as PCR or WB for quantitative measurements [42,43,44].

### 2.7. Histology

Serial 5 µm thickness sections of kidney, previously fixed in paraformaldehyde 4% PBS, were dewaxed in xylene, then they were hydrated in decreasing concentrations of alcohols and stained with the trichrome staining kit according to the manufacturer’s instructions (Masson trichrome Goldner staining kit, Bio-Optica Milano Spa, Milano, Italy). Within each of the experimental groups, a random selection of the sections was carried out for qualitative analysis.

### 2.8. Urinary and renal Oxidative Status Parameters

Quantification of urinary thiobarbituric acid reactive substances (TBARS) was performed as previously reported by Sousa et al. [45]. Urinary 8-isoprostane quantification was performed using a commercial kit according to the protocol provided by the manufacturer (Urinary Isoprostane ELISA Kit; Oxford Bio-medical Research Inc., Oxford, MI, USA). Renal H_2_O_2_ production was quantified in cortex and medulla samples that were incubated for 60 min at 37 °C in 1 mL of oxygenated KREBS-HEPES media. The media were then used for determination of H_2_O_2_ production using a commercial kit according to the protocol provided by the manufacturer (Amplex RedHydrogen Peroxide Assay kit; Molecular Probes, Eugene, OR, USA). The activities of catalase and glutathione peroxidase (GPx) were determined in the supernatant of centrifuged (10 min, 15,700 *g*, 4 °C) homogenized kidney samples (cold phosphate buffer, 50 mM, 7.4, containing Triton 0.1% (*v*/*v*)). Catalase activity was quantified by monitoring H_2_O_2_ decay at 240 nm, at 25 °C during 40 s [46]. One unit of catalase was defined as the amount of enzyme that decomposes 1mmol of H_2_O_2_ per min. Results are expressed as units per mg of protein (extinction coefficient of 0.0394 mM/cm). GPx activity was assayed spectrophotometrically by following NADPH oxidation at 340 nm when glutathione is regenerated by glutathione reductase [46]. Results are expressed as nmol of oxidized NADPH per min per mg of protein (extinction coefficient of 6.22 mM/cm).

### 2.9. Statistical Analysis

Immunostaining is expressed as the percentage of the tissue total area. Results are expressed as the median and 25th–75th percentiles (P25–P75); n denotes the number of animals used in each group. The difference between the treatments, in comparison to the SHR-STZ group, for each receptor’s immunostaining in the renal structures was assessed using the Kruskal–Wallis followed by Dunn’s multiple comparisons test. Furthermore, for each renal structure studied (superficial glomeruli—SG, deep glomeruli—DG, proximal collective tube—PCT, Loop of Henle—LH, distal collecting tube—DCT, and collecting duct—CD), this approach was used to compare the immunostaining against each receptor among the SHR-STZ, SHR-STZ + CADO, and SHR-STZ + DPSPX groups. GraphPad Prism 7 software (San Diego, CA, USA), was used for the statistical analysis, and a *p*-value < 0.05 was considered significant.

## 3. Results

### 3.1. Metabolic Parameters, Renal Function, and Blood Pressure in SHR-STZ Rats Treated with CADO or DPSPX

The SHR-STZ-CADO group had a lower plasma glucose concentration (232 ± 51 vs. 382 ± 44 mg/dL, *p* < 0.05), glucosuria (13.2 ± 0.9 vs. 23.4 ± 2.4 g/kg/24h, *p* < 0.05), proteinuria (84 ± 13 vs. 198 ± 24 mg/kg/24 h, *p* < 0.05), and SBP (114 ± 4 vs. 143 ± 8 mmHg, *p* < 0.05) when compared with the SHR-STZ group (Table S1). The SHR-STZ-DPSPX group had lower FENa^+^ (0.5 ± 0.1 vs. 0.8 ± 0.1%, *p* < 0.05) when compared with the SHR-STZ group (Table S1).

### 3.2. Effect of Endogenous and Exogenous Adenosine on the Distribution Profile and Expression of Adenosine A_1_ and A_2B_ Receptors in SHR-STZ Rats

**Figure 2:**
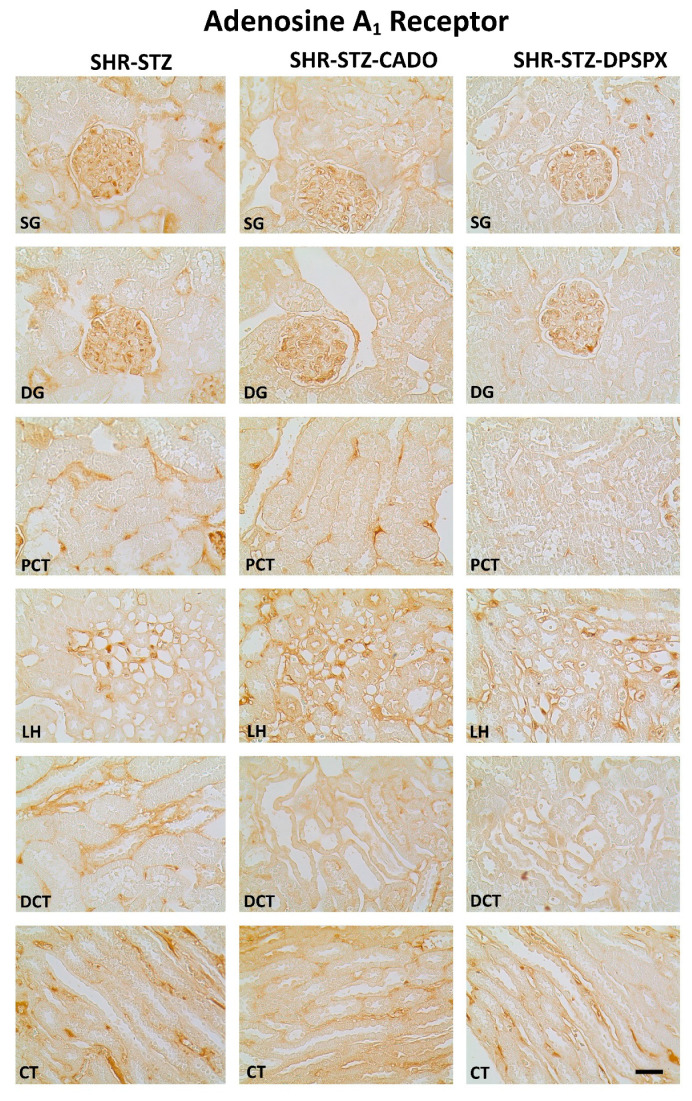
Representative photomicrographs of the immunoreactivity against the adenosine A_1_ receptors in the superficial (SG) and deep glomeruli (DG), proximal convoluted tubule (PCT), distal convoluted tubule (DCT), loop of Henle (LH), and collecting tubule (CT) from kidney sections of SHR-STZ (left panel), SHR-STZ + CADO (middle panel), and SHR-STZ + DPSPX (right panel) animals. Bars: 20 µm.

**Figure 3:**
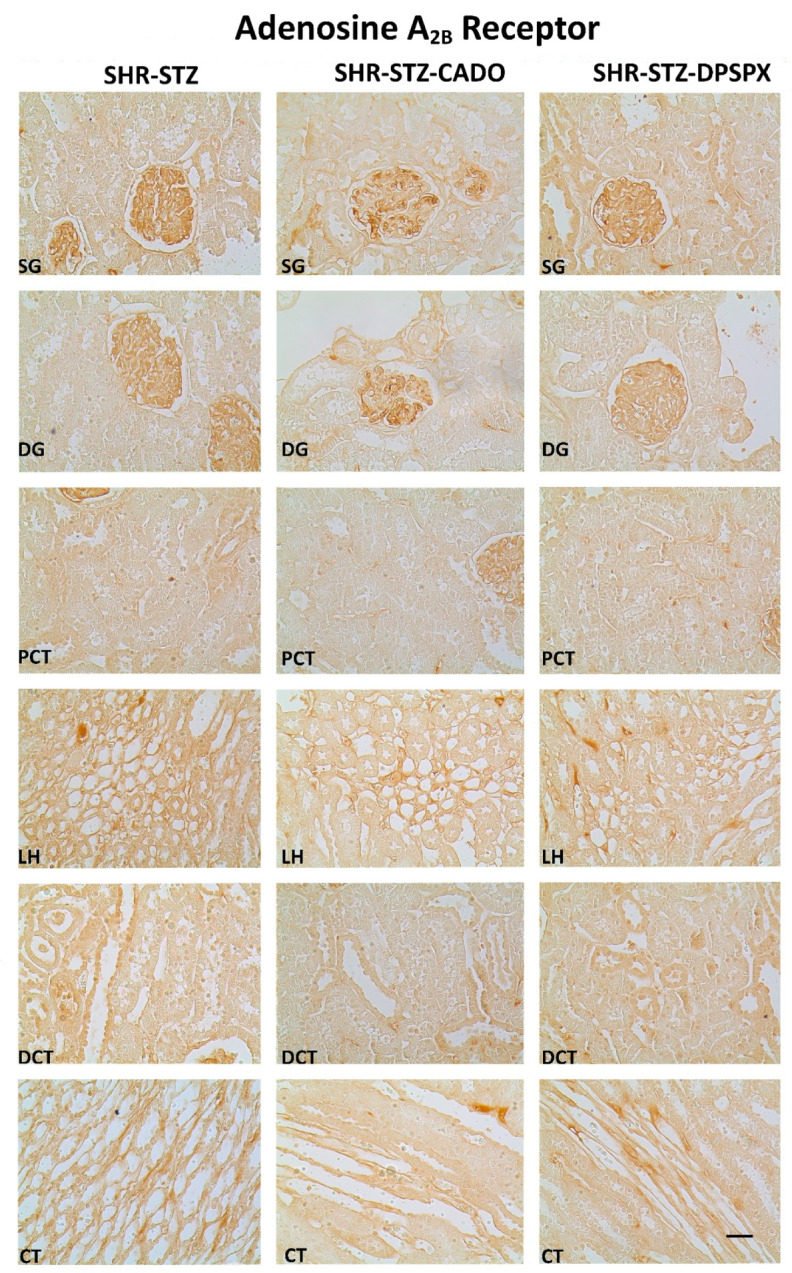
Representative photomicrographs of the immunoreactivity against the adenosine A_2B_ receptors in the superficial (SG) and deep glomeruli (DG), proximal convoluted tubule (PCT), distal convoluted tubule (DCT), loop of Henle (LH), and collecting tubule (CT) from kidney sections of SHR-STZ (left panel), SHR-STZ + CADO (middle panel), and SHR-STZ+DPSPX (right panel) animals. Bars: 20 µm.

**Figure 4:**
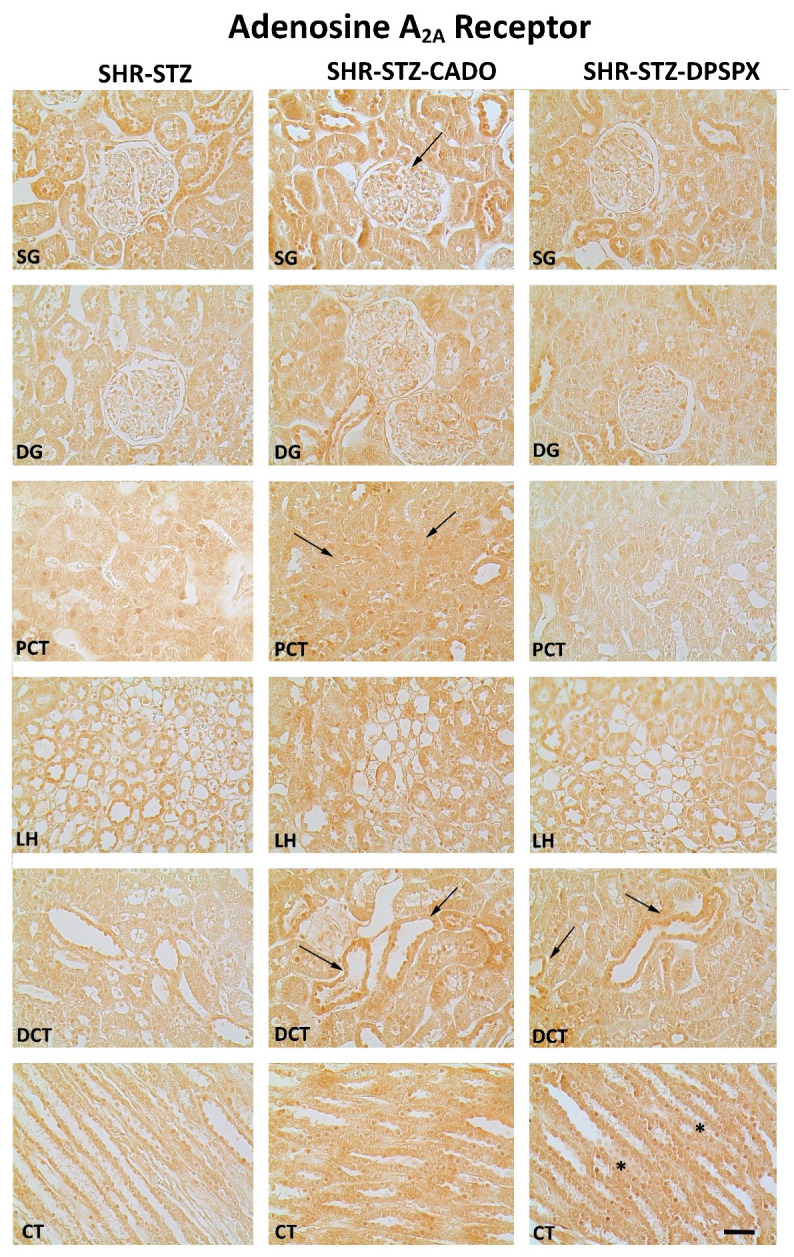
Representative photomicrographs of the immunoreactivity against the adenosine A_2A_ receptors in the superficial (SG) and deep glomeruli (DG), proximal convoluted tubule (PCT), distal convoluted tubule (DCT), loop of Henle (LH), and collecting tubule (CT) from kidney sections of SHR-STZ (left panel), SHR-STZ + CADO (middle panel), and SHR-STZ + DPSPX (right panel) animals. Filled arrows: evidence more pronounced immunoreactivity than that exhibited by the SHR-STZ control group; *: evidence more pronounced immunoreactivity than that exhibited by the SHR-STZ+CADO group. Bars: 20 µm.

**Figure 5:**
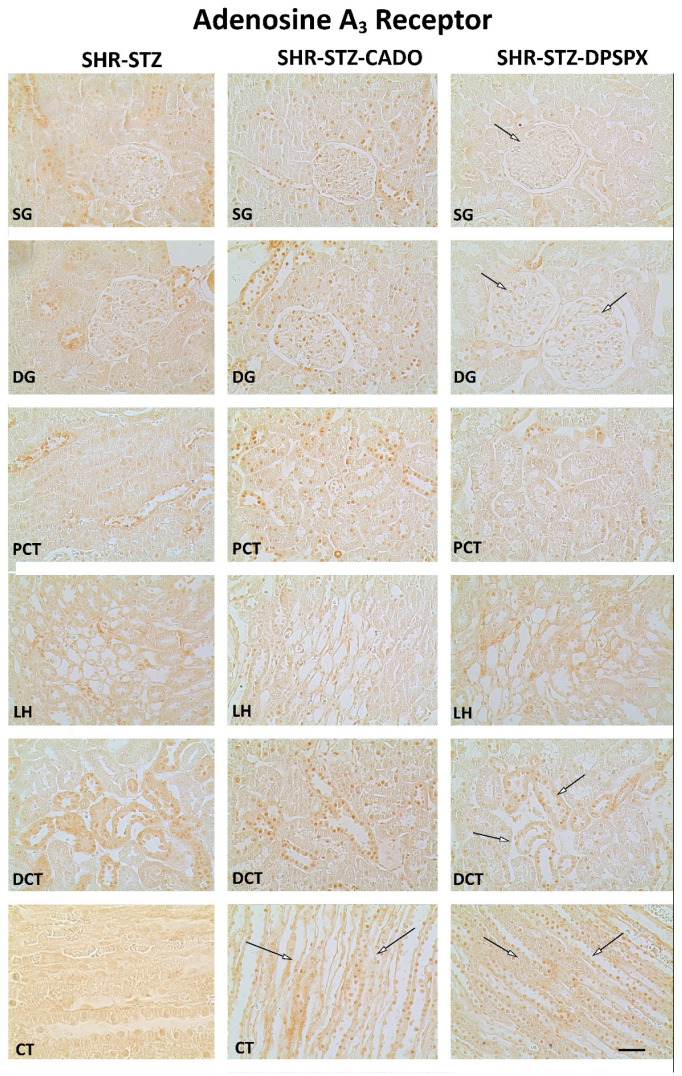
Representative photomicrographs of the immunoreactivity against the adenosine A_3_ receptors in the superficial (SG) and deep glomeruli (DG), proximal convoluted tubule (PCT), distal convoluted tubule (DCT), loop of Henle (LH), and collecting tubule (CT) of SHR-STZ (left panel), SHR-STZ + CADO (middle panel), and SHR-STZ + DPSPX (right panel) animals. Open arrows: evidence less pronounced immunoreactivity than that exhibited by SHR-STZ. Bars: 20 µm.

Immunoreactivity against the four adenosine receptor subtypes (A_1_, A_2A_, A_2B_, A_3_) was observed in all the kidney structures studied: SG, DG, PCT, LH, DCT, and CT in all experimental groups (Figure 2, Figure 3, Figure 4 and Figure 5).

SHR-STZ rats treated with CADO or DPSPX showed similar immunoreactivity against the adenosine A_1_ or A_2B_ receptors in either renal structure studied when compared with the corresponding structure of control SHR-STZ rats (Figure 2 and Figure 3, respectively, and Table 1). We observed a more pronounced expression of the adenosine A_1_ and A_2B_ receptors in the glomeruli (SG and DG) than in the renal tubular structures (PCT, DCT, LH, or CT) of the three experimental groups. However, the immunoreactivity against the adenosine A_1_ receptors was found mainly in mesangial cells, while that against the adenosine A_2B_ receptors was found predominantly in mesangial cells and podocytes (Figure 2 and Figure 3, respectively).

### 3.3. Effect of Endogenous and Exogenous Adenosine on the Distribution Profile and Expression of Adenosine A_2A_ and A_3_ Receptors in SHR-STZ Rats

**Figure 6:**
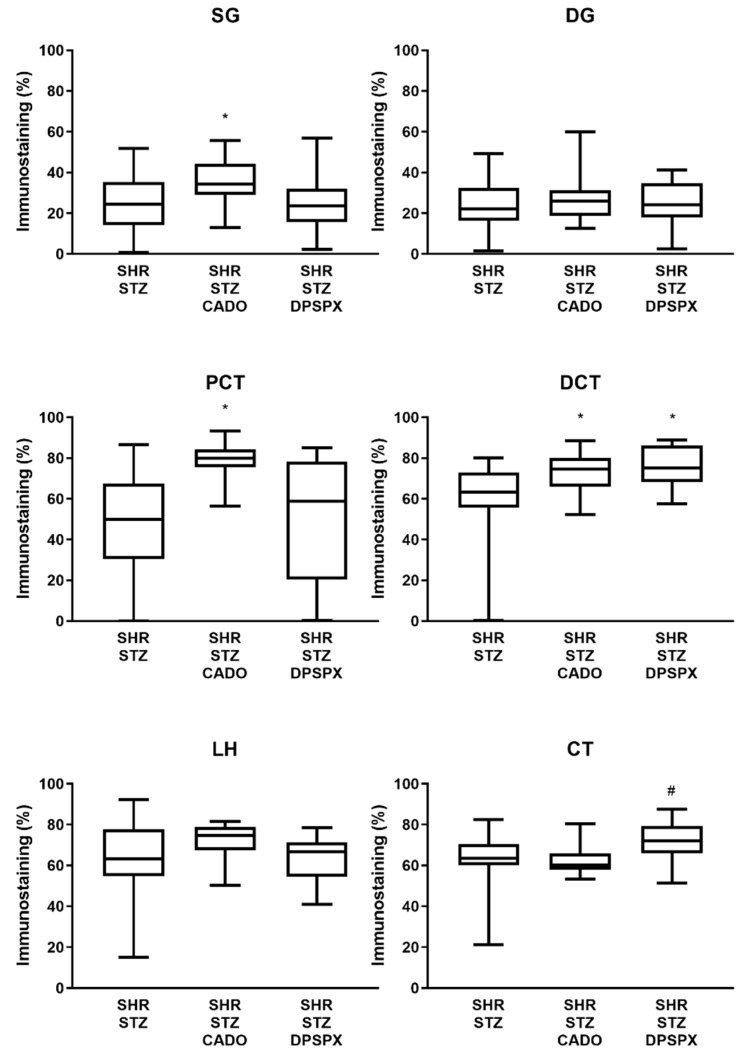
Quantitative analysis of the immunostaining (staining fractional area in percentage of the tissue total area; using the semiautomated computer-assisted image analysis “SACAIA” method) for the adenosine A_2A_ receptors in the six renal structures from SHR-STZ, SHR-STZ + CADO, and SHR-STZ + DPSPX rats. Superficial (SG) and deep (DG) glomeruli, proximal (PCT) and distal (DCT) convoluted tubules, loop of Henle (LH), and collecting tubule (CT). Values are the median and 25th–75th percentiles (P25-P75) from four rats. * *p* < 0.05 vs. the corresponding SHR-STZ group; ^#^ *p* < 0.05 vs. the corresponding SHR-STZ + CADO group.

The expression of the adenosine A_2A_ (Figure 4) and A_3_ (Figure 5) receptors was altered by both CADO and DPSPX treatment. In the SHR-STZ + CADO group, the immunoreactivity against the adenosine A_2A_ receptor was higher in SG (but not in DG), PCT, and DCT than in the correspondent structures of control SHR-STZ rats (Fig4, filled arrows, and Figure 6). Furthermore, SHR-STZ + DPSPX rats showed higher immunoreactivity against the adenosine A_2A_ receptor in DCT than control SHR-STZ rats (Figure 4, filled arrows, and Figure 6). Furthermore, in CT, the immunoreactivity against the adenosine A_2A_ receptor was higher in SHR-STZ+DPSPX rats than in SHR-STZ+CADO rats (Figure 4, asterisk, and Figure 6). The expression of the adenosine A_2A_ receptor was more evident in the tubular structures than in the glomeruli and could be mostly visualized both in the plasma membrane, as well as in the nuclei of tubular cells (Figure 4).

**Figure 7:**
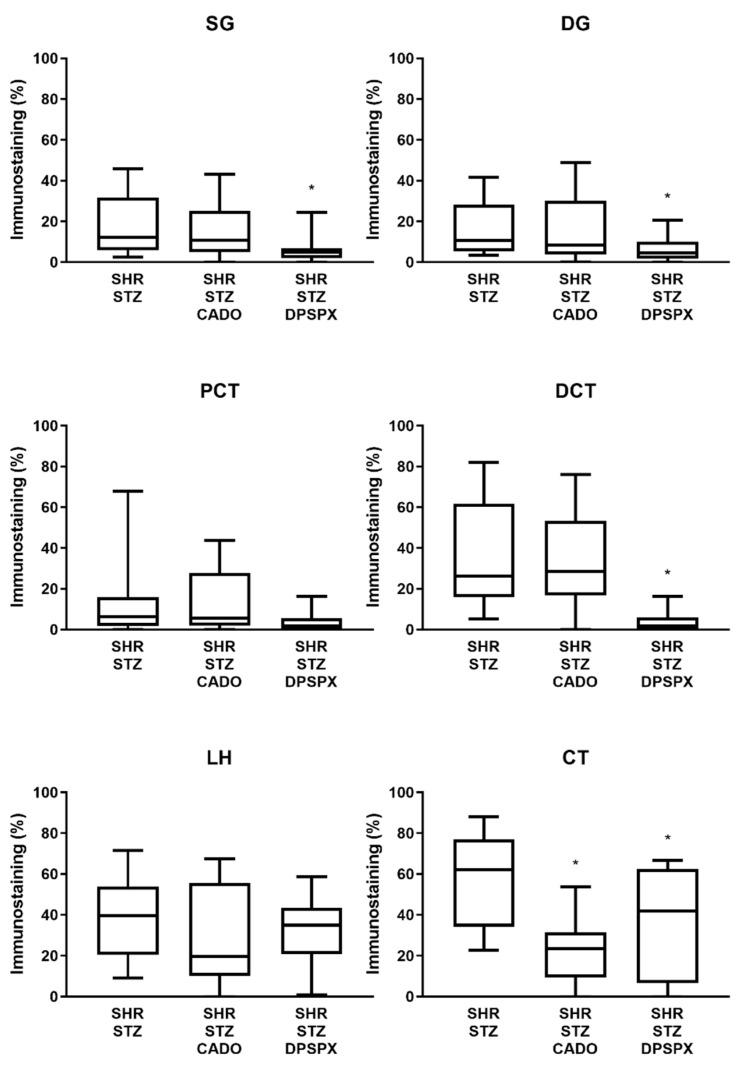
Quantitative analysis of the immunostaining (staining fractional area in percentage of the tissue total area; using the SACAIA method) for the adenosine A_3_ receptors in the six renal structures from SHR-STZ, SHR-STZ+CADO, and SHR-STZ+DPSPX rats. Superficial (SG) and deep (DG) glomeruli, proximal (PCT) and distal (DCT) convoluted tubules, loop of Henle (LH), and collecting tubule (CT). Values are the median and 25th–75th percentiles (P25-P75) from four rats. * *p* < 0.05 vs. the corresponding SHR-STZ group.

Concerning the immunoreactivity against the adenosine A_3_ receptor, it was much weaker than that observed for the other adenosine receptors (Figure 2, Figure 3, Figure 4 and Figure 5). In the SHR-STZ+CADO group, the immunoreactivity against the adenosine A_3_ receptor was lower than that observed in the control SHR-STZ group only in CT (Figure 5, open arrows, and Figure 7). Lower immunoreactivity against the adenosine A_3_ receptor was also observed in the SG, DG, DCT, and CT of SHR-STZ+DPSPX rats compared with control SHR-STZ rats (Figure 5, open arrows, and Figure 7). The immunoreactivity against the adenosine A_3_ receptor was markedly observed in the nuclei of glomerular and tubular cells (Figure 5), and the changes described in the treatment groups were also mostly observed for the immunoreactivity observed in the nuclei of cells (Figure 5).

### 3.4. Effect of Endogenous and Exogenous Adenosine on the Glomeruli of SHR-STZ Rats Treated with CADO or DPSPX

**Figure 8:**
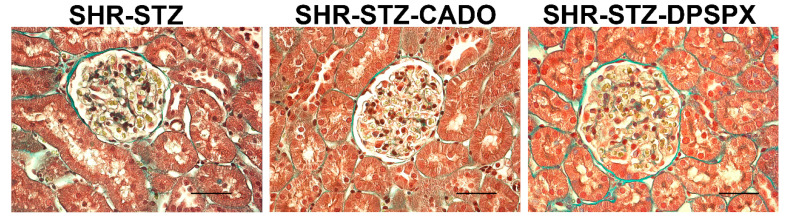
Representative photomicrographs of Masson’s trichrome stain of glomeruli of SHR-STZ (left panel), SHR-STZ + CADO (middle panel), and SHR-STZ + DPSPX (right panel) animals. Bars: 20 µm.

The glomeruli of SHR-STZ + CADO rats showed a thinner inner membrane of Bowman’s capsule and thinner endothelial cells than those observed in SHR-STZ glomeruli (Figure 8). Differently, in the glomeruli of SHR-STZ + DPSPX rats, we observed a thicker inner membrane of Bowman’s capsule, thicker endothelial cells, and higher number of intraglomerular mesangial cells when compared with the glomeruli of SHR-STZ rats (Figure 8).

### 3.5. Urinary and Renal Oxidative Status Parameters

Production of H_2_O_2_ and the activity of H_2_O_2_-neutralizing enzymes in the renal medulla of SHR-STZ rats treated with CADO or DPSPX were similar to those observed in the SHR-STZ group (Table S2). Alternatively, SHR-STZ rats treated with CADO or DPSPX had lower production of H_2_O_2_ in the renal cortex with no alteration of the activity of H_2_O_2_-neutralizing enzymes when compared with SHR-STZ (Table S2). No difference was observed in urinary markers of oxidative stress between groups (Table S2).

## 4. Discussion

Our study highlights the adenosine A_2A_ and A_3_ receptors (and not the adenosine A_1_ or A_2B_ receptors) as putative targets to modify diseased renal function in the context of hypertension and diabetes.

Our study design included three experimental groups of SHR rats: the SHR-STZ group (our control group), the SHR-STZ+CADO group (with which we wanted to exogenously stimulate adenosine receptors besides the tonic endogenous activation), and the SHR-STZ+DPSPX group (to block adenosine receptors and indirectly evaluate the role of tonic endogenous adenosine levels). We previously reported [46] that our control SHR-STZ group showed typical signs of early hyperfiltrating diabetic nephropathy when compared with non-diabetic SHR rats, namely hyperglycemia and glycosuria, decreased body weight, increased food ingestion, polydipsia, polyuria, as well as proteinuria and increased glomerular filtration rate. In the present study, we observed that SHR-STZ rats treated with CADO (but not with DPSPX) for seven days showed higher expression of adenosine A_2A_ receptors in SG, PCT, and DCT than control SHR-STZ rats. Interestingly, this difference was observed in the same renal structures where we previously demonstrated a decrease in the expression of the adenosine A_2A_ receptor associated with the induction of diabetes in SHR when compared with normoglycemic SHR [29]. Furthermore, we previously reported that CADO treatment improved glucose metabolism (decreased hyperglycemia and glycosuria) and renal function (decreased proteinuria) [20]. As pointed out in our previous study, the downregulation of adenosine A_2A_ receptors in SHR-STZ rats (when compared with control non-diabetic SHR) [29] could be contributing to the observed increase in intraglomerular pressure leading to hyperfiltration and to the decrease in sodium reabsorption leading to diuresis and natriuresis. In the present study, we also show that SHR-STZ rats treated with CADO have less collagen deposition when compared with that observed in control SHR-STZ rats. Masson’s trichrome staining is an efficient and reproducible method to evaluate fibrosis with functional correlation [47]. As such, treatment with CADO is also associated with decreased renal fibrosis, which can be a result of increased anti-fibrotic adenosine A_2A_ receptors [4,48]. Taken together, these studies of our group, performed in the same experimental conditions, show that in SHR, induction of diabetes with STZ decreases the expression of renal adenosine A_2A_ receptors [29] and that exogenously stimulating adenosine receptors can partially restore that lessened expression of the adenosine A_2A_ receptor. This positive modulatory role of exogenous adenosine (here mimicked by CADO infusion) seems to be specific for the adenosine A_2A_ receptor since we observed no alteration in the expression of A_1_, A_2B_, or A_3_ receptors in these renal structures. The improved expression of adenosine A_2A_ receptors can also represent an important achievement for the control of increased intraglomerular pressure and GFR previously observed in SHR-STZ diabetic rats [46]. In fact, higher expression of adenosine A_2A_ receptors could decrease intraglomerular pressure and increase Na^+^ reabsorption [49,50], thus contributing to attenuated early diabetic hyperfiltration [46]. However, SHR-STZ rats had increased GFR [46], and treatment with CADO had no effect on GFR or fractional excretion of sodium (FE_Na+_), although it lowered proteinuria [20]. Possible explanations for this are the improvement of the basement membrane integrity, which may be a result of the lower SBP observed in this group.

Differently, treating SHR-STZ rats with either CADO or DPSPX increased the expression of the A_2A_ receptor in DCT and decreased the expression of the A_3_ receptor in CT. This suggests that the effect of adenosine on the expression of these receptor subtypes in DCT and CT might be biphasic. Lower endogenous adenosine levels decrease the expression of the A_2A_ receptor, but increase that of the A_3_ receptor (both effects blocked by DPSPX), while higher adenosine levels (mimicked by CADO activation of adenosine receptors over tonic activation by endogenous levels) increase the expression of the A_2A_ receptor, but decrease that of the A_3_ subtype. The increase in the expression of A_2A_ receptor observed in distal nephron (DCT) might contribute to reducing cardiovascular risk by resetting Mg^2+^ levels [51,52].

DPSPX is a non-selective antagonist of adenosine receptors, but it has very low affinity for the A_3_ receptor subtype [53]. In our experimental conditions, the adenosine A_1_, A_2A_, and A_2B_ receptors are blocked by DPSPX, but the A_3_ receptor is free for ligand binding. Therefore, our results with the SHR-STZ+DPSPX group may reflect the role of endogenous adenosine resulting from the activation of adenosine A_3_ receptors. We showed that SHR-STZ+DPSPX rats have similar glucose metabolism, renal function, and SBP to those observed in SHR-STZ rats and similar renal oxidative stress status compared to SHR-STZ+CADO rats. However, the glomeruli of SHR-STZ+DPSPX rats had marked fibrosis when compared with the control SHR-STZ group. The expression and function of the adenosine A_3_ receptor in kidney has seldom been studied, but it has been suggested to delay the development of glomerulosclerosis, due to mesangial cell apoptosis [11]. However, our results are in line with more recent studies implicating the adenosine A_3_ receptor in renal fibrosis. Indeed, molecules with targeted adenosine A_3_ antagonistic activity are being advocated for their renal anti-fibrotic effects [54] including in diabetic nephropathy [55].

Although the expression of the A_3_ receptor was globally low, in vivo treatment with DPSPX further decreased it (almost abolished) in glomeruli, DCT, and CT. This could mean that endogenous levels of adenosine (acting on adenosine A_1_, A_2A_, and/or A_2B_ receptors) are required for its expression, at least in a hypertensive-diabetic condition. Alternatively, the adenosine A_3_ receptor might be downregulated [56] as a result of prolonged stimulation by endogenous adenosine, which is directed to activate this receptor subtype under the blockade of the A_1_, A_2A_, and A_2B_ receptors by DPSPX. This downregulation could be an important mechanism to control adenosine A_3_ receptor-mediated renal fibrosis. This putative fine-tuning regulation by adenosine levels seems to be auto-regulated, since a marked stimulation of adenosine receptors (here represented by the SHR-STZ+CADO group) does not further increase the expression of the A_3_ receptor subtype. Interestingly, in normotensive animals, STZ-induced diabetes increased renal adenosine levels [18] and did not alter adenosine A_3_ receptor mRNA in the kidney [22], although it increased membrane-associated A_3_ protein levels in the renal cortex and decreased it in the renal medulla [22]. Differently, in SHR, induction of diabetes with STZ did not alter the expression of the membrane-bound adenosine A_3_ receptor [29], suggesting that the modulatory role of adenosine on its receptors might also depend on the health condition of the animal (diabetic vs. hypertensive-diabetic). Therefore, one could speculate that in hypertensive SHR-STZ diabetic rats, endogenous levels of adenosine would be beneficial to maintain a proper A_3_-mediated control of glomerular expansion. However, in conditions that favor overactivation of the A_3_ receptor subtype, such as inflammatory conditions [57] and cancer [58], deleterious fibrotic mechanisms would be activated, and an alternative mechanism to control intraglomerular pressure and GFR would be needed; that could be the increase in the expression of the adenosine A_2A_ receptor.

The expression of the adenosine A_1_ and A_2B_ receptors observed in the SHR-STZ group was not different from that observed in the SHR-STZ-CADO or SHR-STZ-DPSPX group. Although the adenosine A_1_ and A_2B_ receptors are abundant in the kidney of hypertensive SHR-STZ diabetic rats, particularly in glomeruli, tonic endogenous or exogenous adenosine levels and/or receptor stimulation do not modify their expression. While our study is on an experimental model (the hypertensive (SHR) and diabetic (STZ-induced) rat), it draws attention of translational relevance for human diabetes associated with hypertension since these patients might not be as sensible as expected to the diuretic/natriuretic effects that have been attributed to selective adenosine A_1_-receptor antagonists [59]. Furthermore, the concentrations of adenosine seen in the diabetic condition are probably high enough to reach the low-affinity A_2B_ receptor [18], and even considering the same level of receptor expression, this might contribute to the progression of hypertensive-diabetic renal damage. Indeed, although the activation of the adenosine A_2B_ receptors might have beneficial effects at early and acute stages of renal damage due to immunomodulatory and anti-inflammatory tissue repair [7] and restrain mesangial cell growth [8], it has been mostly associated with deleterious production of glomerular extracellular matrix, TGB-β1 [18], and VEGF [9,60], leading to glomerulosclerosis [18].

A major strength of our study is that it was focused on the experimental hypertensive-diabetic conditions and not each individual pathology. Furthermore, we defined the distribution profile of the four adenosine receptor subtypes in six different structures of the kidney and were able to distinguish the intracellular distribution. This is relevant since we were able to determine that the effect of adenosine levels on the expression of its own membrane-bound receptors depends not only on the receptor subtype, but also on the kidney structure. Moreover, the immunohistochemistry study allows for the identification/quantification of the receptor protein along with its localization/distribution in the cell/tissue. This is in contrast with experimental molecular approaches like polymerase chain reaction (PCR) or Western blot, which do not recognize proteins in their membrane-bound quaternary structure and do not allow identifying the exact location of the target in a tissue/structure.

A putative limitation of our study is the phenomena of agonist-induced receptor desensitization and downregulation due to internalization, which is described for the four adenosine receptor subtypes [61,62]. Internalization is a slow process, particularly for the adenosine A_1_ receptor [63,64,65], but the treatment with CADO lasted for seven days, giving enough time for internalization of any of the four receptor subtypes to occur. However, the present results were not in line with this rationale, which might be explained by the methodological approach selected for this and other studies. Alternative methodological approaches like PCR or Western blot provide insight on possible downregulation of any of the four adenosine receptors. However, the main aim of the present study was to evaluate the regulatory role of endogenous and exogenous adenosine in the expression of its own membrane receptors. Immunohistochemistry allows for the morphologic description and morphometric quantification of the four adenosine receptors in six renal structures. This could not be obtained with RNA measurements by PCR or protein quantification by Western blot analysis, which would only provide a global characterization of the renal expression of the receptors. Neither immunohistochemistry, nor Western blot, nor PCR allow concluding on the functional status of the receptors under study. However, we observed alterations in renal fibrosis between the experimental groups, and we reported that CADO treatment (but not DPSPX treatment) ameliorated the metabolic profile of SHR-STZ rats, although both CADO and DPSPX treatments attenuated the renal cortical production of H_2_O_2_.

## 5. Conclusions

Taken together, our results show that adenosine has a protective role in hypertensive-DN through the dynamic expression of its receptors, particularly the adenosine A_2A_ and A_3_ receptors. This conclusion is grounded in several arguments. First, the treatment of SHR-STZ animals with CADO ameliorated glucose metabolism (decreased hyperglycemia and glycosuria), renal function (decreased proteinuria), and renal fibrosis (decreased collagen staining in the renal glomeruli), as well as renal oxidative stress (decreased cortical production of H_2_O_2_). Second, this protection is associated with an upregulation of the adenosine A_2A_ receptors, which are renoprotective. Third, blocking adenosine receptors with DPSPX (reflecting tonic activation of the adenosine A_3_ receptors) aggravated renal fibrosis (increased collagen staining in renal glomeruli), although it did not alter metabolic status and renal function except for a decrease in FENa^+^ and renal cortical production of H_2_O_2_. Fourth, this putative prolonged tonic activation of A_3_ receptors appears to induce their downregulation, which could represent a regulatory mechanism to control further renal fibrosis. These results highlight the importance of adenosine A_2A_ upregulation and/or A_3_ receptors’ downregulation as a promising therapeutic target for hypertensive-DN.

## Figures and Tables

**Table 1:** Immunostaining (% of the tissue total area) for A_1_ and A_2B_ adenosine receptors in the renal structures from SHR rats of the three experimental groups.

		SHR-STZ Control	SHR-STZ + CADO	SHR-STZ + DPSPX
**Adenosine A_1_ receptor**				
	**SG**	73.73 (59.53–80.82)	59.30 (41.53–71.27)	68.40 (43.68–76.85)
	**DG**	54.31 (27.32–68.69)	43.36 (28.18–62.38)	48.46 (23.23–65.24)
	**PCT**	28.05 (8.82–54.82)	17.71 (9.35–37.98)	17.11 (6.71–41.13)
	**DCT**	48.04 (23.43–63.49)	52.43 (34.12–65.90)	53.86 (27.15–64.80)
	**LH**	47.24 (39.53–58.24)	52.04 (40.39–59.67)	47.38 (38.20–59.76)
	**CT**	49.71 (42.95–61.29)	47.54 (33.40–53.83)	48.31 (41.70–58.19)
**Adenosine A_2B_ receptor**				
	**SG**	74.94 (61.10–80.16)	72.91 (68.24–76.89)	72.07 (62.75–77.70)
	**DG**	69.02 (60.16–74.04)	72.47 (66.47–74.48)	65.83 (58.95–73.72)
	**PCT**	8.06 (1.82–42.92)	17.74 (10.89–29.38)	14.01 (6.77–23.34)
	**DCT**	52.38 (21.83–69.54)	51.76 (42.08–60.35)	40.42 (26.19–62.96)
	**LH**	39.81 (33.22–50.03)	44.40 (41.29–46.54)	41.03 (34.66–47.14)
	**CT**	45.74 (34.95–57.54)	43.78 (41.67–51.64)	44.00 (36.38–54.81)

Values are the median (P25-P75). SG = superficial glomeruli; DG = deep glomeruli; PCT = proximal convoluted tubule; DCT = distal convoluted tubule; LH = loop of Henle; CT = collecting tubule.

## Supplementary Materials

Supplementary Materials can be found at https://www.mdpi.com/2227-9059/8/11/529/s1: Table S1: Metabolic and renal function parameters of SHR-STZ rats treated with CADO or DPSPX., Table S2: Production of hydrogen peroxide (H_2_O_2_) and activity of H_2_O_2_-neutralizing enzymes in the kidney tissue and urinary markers of oxidative stress of SHR-STZ rats treated with CADO or DPSPX.
