# Supplementary material for: Adenosine A2A and A3 Receptors as Targets for the Treatment of Hypertensive-Diabetic Nephropathy
Source: Biomedicines. 2020 Nov 23;8(11):529. doi: 10.3390/biomedicines8110529 (PMC7700226; doi:10.3390/biomedicines8110529)
Supplement: Supplementary file 1 [file biomedicines-08-00529-s001.pdf]

Table S1. Metabolic and renal function parameters of SHR-STZ rats treated with CADO or DPSPX.

	P-glucose mg/dL	BW g	Food intake g/24h	Water intake mL/24h	U-excretion mL/24h	U-glucose g/kg/24h	U-proteins mg/kg/24h	GFR mL/min	FE_{Na} %	SBP mmHg
**SHR-STZ**	382 ± 44	177 ± 9	25.3 ± 1.3	126 ± 9	106 ± 10	23.4 ± 2.4	198 ± 24	2.0 ± 0.3	0.8 ± 0.1	143 ± 8
**SHR-STZ+CADO**	232 ± 51*	196 ± 7	24.7 ± 1.0	115 ± 6	96 ± 5	13.2 ± 0.9*	84 ± 13*	1.6 ± 0.1	0.7 ± 0.1	114 ± 4*
**SHR-STZ+DPSPX**	271 ± 63	174 ± 6	24.3 ± 1.8	109 ± 10	90 ± 10	22.1 ± 2.1	142 ± 15	1.7 ± 0.2	0.5 ± 0.1*	137 ± 8

Abbreviations: P-plasma; BW-body weight; U-urine; GFR-glomerular filtration rate; FE_{Na} - fractional excretion of Na^+; SBP – Systolic blood pressure. Results expressed as mean ± S.E.M.. * $p < 0.05$ vs SHR-STZ. Data concerned with SHR-STZ and SHR-STZ+CADO groups has been partially published elsewhere[20].

Table S2. Production of hydrogen peroxide (H_2O_2) and activity of H_2O_2-neutralizing enzymes in the kidney tissue and urinary markers of oxidative stress of SHR-STZ rats treated with CADO or DPSPX.

	Renal Medulla			Renal Cortex			Urine		
	**H_2O_2 production nmol/mg prot**	**GPX activity nmol NADPH oxid/min/mg prot**	**Catalase activity Ucat/mg prot**	**H_2O_2 production nmol/mg prot**	**GPX activity nmol NADPH oxid/min/mg prot**	**Catalase activity** Ucat/mg prot	**Isoprostanes** ng/24h/kg	**TBARS** μmol/24h/Kg	**H_2O_2** μmol/24h/Kg
**SHR-STZ**	0.9 ± 0.1	95.0 ± 8.3	47.9 ± 5.4	0.8 ± 0.1	313.2 ± 30.2	29.7 ± 1.8	454.7 ± 38.8	5.0 ± 0.6	12.5 ± 2.0
**SHR-STZ CADO**	0.7 ± 0.1	82.3 ± 9.0	48.2 ± 5.4	0.4 ± 0.1*	299.5 ± 29.6	37.7 ± 4.9	453.8 ± 77.9	4.3 ± 0.3	8.7 ± 1.5
**SHR-STZ DPSPX**	0.7 ± 0.1	82.2 ± 10.3	34.9 ± 3.8	0.5 ± 0.1*	304.2 ± 30.1	33.7 ± 2.8	351.8 ± 58.9	4.7 ± 0.1	20.8 ± 3.3

Abbreviations: GPX- glutathione peroxidase; TBARS- thiobarbituric acid reactive substances. Results expressed as mean ± S.E.M.. * $p < 0.05$ vs SHR-STZ. Data concerned with SHR-STZ and SHR-STZ+CADO groups has been partially published elsewhere[20].
